# Clear and Consistent Imaging of Hippocampal Internal Architecture With High Resolution Multiple Image Co-registration and Averaging (HR-MICRA)

**DOI:** 10.3389/fnins.2021.546312

**Published:** 2021-02-11

**Authors:** Lawrence Ver Hoef, Hrishikesh Deshpande, Joel Cure, Goutham Selladurai, Julia Beattie, Richard E. Kennedy, Robert C. Knowlton, Jerzy P. Szaflarski

**Affiliations:** ^1^Department of Neurology, The University of Alabama at Birmingham, Birmingham, AL, United States; ^2^Department of Biomedical Engineering, The University of Alabama at Birmingham, Birmingham, AL, United States; ^3^Neurology Service, Birmingham VA Medical Center, Birmingham, AL, United States; ^4^Department of Radiology, The University of Alabama at Birmingham, Birmingham, AL, United States; ^5^Division of Behavioral Medicine and Clinical Psychology, Cincinnati Children’s Hospital Medical Center, Cincinnati, OH, United States; ^6^Division of Gerontology, Geriatrics, and Palliative Care, Department of Medicine, The University of Alabama at Birmingham, Birmingham, AL, United States; ^7^Department of Neurology, University of California, San Francisco, San Francisco, CA, United States

**Keywords:** hippocampus, subfields, high-resolution MRI, internal architecture, dentation

## Abstract

Magnetic resonance imaging of hippocampal internal architecture (HIA) at 3T is challenging. HIA is defined by layers of gray and white matter that are less than 1 mm thick in the coronal plane. To visualize HIA, conventional MRI approaches have relied on sequences with high in-plane resolution (≤0.5 mm) but comparatively thick slices (2–5 mm). However, thicker slices are prone to volume averaging effects that result in loss of HIA clarity and blurring of the borders of the hippocampal subfields in up to 61% of slices as has been reported. In this work we describe an approach to hippocampal imaging that provides consistently high HIA clarity using a commonly available sequence and post-processing techniques that is flexible and may be applicable to any MRI platform. We refer to this approach as High Resolution Multiple Image Co-registration and Averaging (HR-MICRA). This approach uses a variable flip angle turbo spin echo sequence to repeatedly acquire a whole brain T2w image volume with high resolution in three dimensions in a relatively short amount of time, and then co-register the volumes to correct for movement and average the repeated scans to improve SNR. We compared the averages of 4, 9, and 16 individual scans in 20 healthy controls using a published HIA clarity rating scale. In the body of the hippocampus, the proportion of slices with good or excellent HIA clarity was 90%, 83%, and 67% for the 16x, 9x, and 4x HR-MICRA images, respectively. Using the 4x HR-MICRA images as a baseline, the 9x HR-MICRA images were 2.6 times and 16x HR-MICRA images were 3.2 times more likely to have high HIA ratings (*p* < 0.001) across all hippocampal segments (head, body, and tail). The thin slices of the HR-MICRA images allow reformatting in any plane with clear visualization of hippocampal dentation in the sagittal plane. Clear and consistent visualization of HIA will allow application of this technique to future hippocampal structure research, as well as more precise manual or automated segmentation.

## Introduction

**FIGURE 1:**
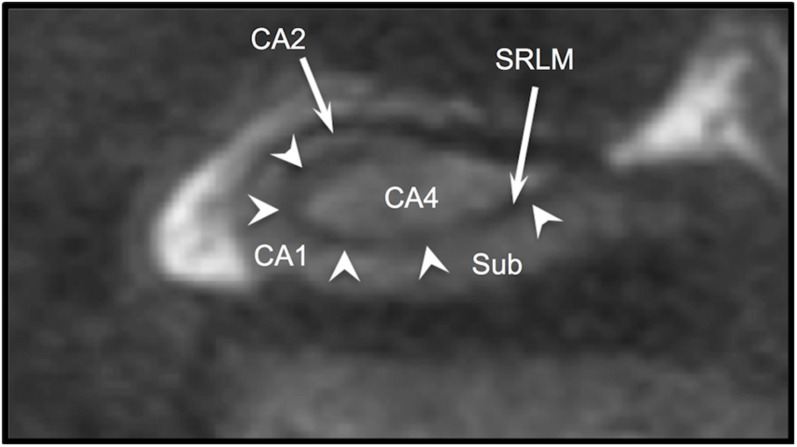
T2w coronal section through body of hippocampus. Arrowheads indicate the “dark band” of the SRLM. Sub (subiculum), CA1, CA2, and CA4 indicate regions of Ammon’s horn.

The hippocampus is one of the most studied subcortical structures in the brain. It has been linked to the pathobiology of epilepsy (Sloviter, 1987; De Lanerolle et al., 1989; Wieser, 2004), Alzheimer’s disease (Jack et al., 1992), schizophrenia (Lahti et al., 2006; Kraguljac et al., 2013, 2016), PTSD (Smith, 2005; Shin et al., 2006; Wang et al., 2010), and TBI (Ariza et al., 2006). For many years, hippocampal imaging research focused largely on volumetric measurements and surface morphometry, but in recent years there has been increasing interest in studying specific hippocampal subfields (Mueller et al., 2007; Van Leemput et al., 2009; Yushkevich et al., 2010, 2015a; Pluta et al., 2012; Wisse et al., 2012, 2016). A number of protocols for manual subfield segmentation have been proposed and widely discussed (Zeineh et al., 2001; Mueller et al., 2007; La Joie et al., 2010; Malykhin et al., 2010; Wisse et al., 2012; Winterburn et al., 2013; Yushkevich et al., 2015a; Steve et al., 2017), and several automated subfield segmentation software tools are available (Van Leemput et al., 2009; Pipitone et al., 2014; Iglesias et al., 2015; Yushkevich et al., 2015b). Precise subfield segmentation requires direct visualization of the hippocampal internal architecture, defined by apposing layers of gray and white matter that create the characteristic spiral appearance of Ammon’s horn in coronal section. Specifically, the strata radiatum, lacunosum, and moleculare (SRLM) together have a hypointense (dark) appearance on T2w scans, while the pyramidal cell layer (CA1-4) is more hyperintense and is isointense with cortical gray matter (Figure 1). However, the dark band of the SRLM is often not clearly or consistently seen in images acquired with conventional MRI sequences. We have previously demonstrated that hippocampal internal architecture (HIA) is seen clearly in only 39% of slices through the body of the hippocampus using common high-resolution T2-weighted coronal images (Ver Hoef et al., 2013b), and in fact even adjacent slices from a good-quality scan may show internal architecture with drastically different clarity. As a consequence, manual subfield segmentation in some slices must rely on *inferring* the boundaries of Ammon’s horn based on “fuzzy” image features or expected boundary location as opposed to direct, clear visualization of the SRLM in each slice. Likewise, many of the automated methods must rely heavily on atlas/template-based approaches and somewhat less on the boundary information contained in the image itself (Wisse et al., 2014). Consequently the resulting automated segmentation may reflect the template to a greater or lesser degree than the target image.

Standard high-resolution sequences with adequate in-plane resolution (<0.5 mm) typically require a slice thickness of 2–3 mm or greater to have adequate signal-to-noise ratio (SNR). These relatively thick slices result in volume averaging of any features that vary through the thickness of a single slice. In some cases, the gray and white matter layers may be very consistent across the slice thickness resulting in clear visualization of internal architecture, while in others slices the layers may vary or undulate through the thickness of the slice resulting in blurring of these features (i.e., volume averaging effects). Sequences with relatively thinner slices, like common T1-weighted volumetric sequences with ∼1 mm isotropic resolution, lack the resolution in the coronal plane to fully depict the contours of the SRLM and Ammon’s horn, which may be less than 1 mm thick. If we simply modify a typical 2D T2w TSE sequence to have thinner slices, the time of acquisition (TA) doubles for each reduction of slice thickness by half. So a 6-min scan with 3 mm thick slices would become a 12-min scan with 1.5 mm thick slices, which dramatically increases sensitivity to movement. Indeed experience teaches us that even highly motivated control subjects have a hard time remaining still enough for a 10-min scan, so simple extension of a 2D TSE sequence cannot work in this case. Further, the SNR, which is directly related to the quality of the image, drops in half when slice thickness is cut in half, resulting in a noisy image that precludes confident delineation of subtle image features such as the margins of the SRLM and hippocampal subfields. Poor SNR can be improved by acquiring multiple samples and averaging them (Bronen et al., 2002), but SNR only improves with the square root of the increase in the number of samples averaged, so to double the SNR requires increasing the number of samples by a factor of 4 and tripling the SNR requires increasing the samples by a factor of 9. The dramatically increased time this takes would absolutely necessitate compensating for the inevitable movement associated with long scanning sessions. From these considerations we can conclude that any sequence that will show hippocampal internal architecture clearly must have the following characteristics: sufficient resolution in the coronal plane to show the SRLM and boundaries of Ammon’s horn clearly; sufficiently thin slices (i.e., resolution in the A-P direction) to minimize volume averaging; and the TA of a single acquisition must be reasonable so as to keep movement artifacts to a minimum in most subjects.

In this work, as proof-of-principle, we describe a flexible approach to clearly and consistently image HIA. Unlike some advanced approaches that require ultra-high field strength (e.g., 7T), special research pulse sequences, or complicated post-processing pipelines, we use a first generation clinical 3T platform with a basic 8-channel parallel coil with a commonly available sequence. The approach uses at variable flip-angle turbo spin echo sequence (BrainView, Phillips Healthcare) to acquire a 3D volumetric T2-weighted image with parameters adjusted for the shortest possible TA while preserving gray-white contrast, but with relative disregard for SNR. By acquiring an image volume in as short TA as possible, we minimize movement artifact in the individual scan, then we repeat the scan many times and co-register each scan to the first scan in post-processing using freely available software. The co-registered scans are then averaged to improve SNR. We refer to this approach as HR-MICRA (High Resolution Multiple Image Co-registration and Averaging). While the concept of co-registering and averaging multiple scans to improve SNR is nothing new (Bronen et al., 2002), the innovation of this work lies in the combination of the specific application to HIA visualization, the counter-intuitive use of low SNR base images, and the absence of necessity of sophisticated hardware or proprietary software. We report the performance of the HR-MICRA approach in regard to depicting HIA clarity at three different levels of averaging (4x, 9x, and 16x), using a single acquisition of a common high-resolution 2D T2-weighted scan as a baseline reference. The purpose of this work is to demonstrate that it is possible to show HIA with good to excellent clarity in a high proportion of slices using relatively basic equipment and methods with this approach.

## Materials and Methods

### Subject Demographics

After obtaining approval from our Institutional Review Board, 20 healthy control subjects were recruited, consented, and enrolled. Among study subjects, 11 were female, and ages ranged from 21 to 57 with a mean of 28.25 years. Four participants self-identified as African-American, 15 as white, and one as “other.” Years of formal education ranged from 12 to 22 years with a mean of 16.

### Imaging Sequences

All scans were performed on a Philips Achieva 3T platform with an 8-channel head coil. The HR-MICRA scans were based on a variable flip angle turbo spin echo sequence [BrainView on the Phillips platform, similar to SPACE (Sampling Perfection with Application optimized Contrasts using different flip angle Evolution) on Siemens platforms or CUBE on GE platforms]. By using a prescribed evolution of refocusing pulses with variable flip angles, this sequence preserves nearly constant T2 contrast between gray and white matter over a prolonged echo train duration allowing for over 100 echoes in a single TR (Busse et al., 2006). Since almost all of these refocusing pulses are less than 180°, long echo trains can be used without exceeding SAR limits (Busse et al., 2006) allowing for efficient acquisition of 3D T2-weighted data. While most structural imaging sequences are designed to balance TA, resolution, and SNR, this sequence was designed specifically to minimize TA for a specific target resolution while preserving *contrast* as opposed to SNR. A typical T2 sequence uses a moderately long TE (100–140 ms) and a very long TR (3000–4000 ms) to maximize longitudinal relaxation prior to the next excitation pulse. One can easily obtain high resolution within a long TR by extending the echo train length further into the TR, but this has the consequence of prolonging the effective TE, which ultimately decreases the T2 contrast between gray and white matter. This results in an image in which tissue is dark and CSF is bright, which works very well for high-resolution imaging of CSF filled structures like the semi-lunar canals and cochlea of the inner ear, but gray-white borders become indistinct. Variable flip angle sequences help with this effect dramatically by maintaining a shorter equivalent TE for a given effective TE, Busse et al. (2006) but there is a limit to this ability. In light of this, we designed our sequence with an equivalent TE that provides good gray-white contrast (143 ms), but shortened the TR dramatically from a typical TR of 3200 to 1750 ms. This results in decreased SNR due to the fact that the transverse magnetization has not fully relaxed into the longitudinal axis, but it also shortens the scan time to a reasonable amount that most subjects can remain still for. Conversely, using a more typical TR (e.g., 3200 ms), each scan would be over 11 min, but few subjects can remain still for that long.

The time-optimized sequence has an in-plane resolution of 0.5 mm in the coronal plane and a slice thickness of 0.75 mm (A-P) with a scan time of 6:02 min. The resolution of 0.5 mm in the coronal plane was chosen to ensure that HIA was visible throughout the length of the SRLM given that the thickness of this band may be slightly less than 1 mm. The slice thickness of 0.75 mm was secondarily derived as the minimum resolution that could be acquired with a target time of acquisition of approximately 6 min to minimize the likelihood of intra-scan movement. We chose to use this non-isotropic resolution with lower resolution in the A-P direction instead of a larger isotropic voxel size because curvature of the SRLM in A-P direction is generally less than in the coronal plane, and we wanted to maintain high resolution in the plane in which HIA is visualized. This is a whole-brain sequence with parameters as follows: FOV (mm) = FH 200/RL 178/AP 219, TR (ms) = 1750, TE_*eff*_/TE_*equiv*_ (ms) = 348/143, TSE factor = 100, Echo spacing (ms) = 6.0, and Echo train duration (ms) = 643. This sequence was repeated 16 times over one long session for most subjects. For two subjects, the scan was broken into two shorter sessions due to subject and scanner availability. There was no perceptible difference in the scan-to-scan variation between individual scans acquired across two sessions as compared to those acquired within a single session. The total scan time for all 16 iterations of the sequence is 96.5 min, not including brief pauses between scans to check on patient comfort. In post-processing, the scans were co-registered using FSL-FLIRT (Jenkinson et al., 2002) and averaged. A shell script to perform co-registration and averaging along with detailed instructions are included in the Supplementary Material. To assess the effect of the number of scans averaged on image quality, mean images were generated from a subset of 4 and 9 scans as well as the total 16 scans (HR-MICRA 4x, HR-MICRA 9x, and HR-MICRA 16x), which reflect a theoretical improvement in SNR by a factor of 2, 3, and 4, respectively, over a single base scan. In a few subjects, one to four scans were discarded due to obvious, marked intra-scan movement artifact such that inclusion in the average would degrade image quality. Scans with minor movement artifact were not excluded. One subject had one scan removed, two subjects had two scans removed, one subject had three scans removed, and three subjects had four scans removed. The removed scans tended to be at the end of the scanning session, presumably due to the fact that some subjects were becoming restless due to the long scanning session. In cases where scans were removed due to movement, the remaining scans were averaged and used in place of the full set of 16 scans in reporting the proportions of slices with each HIA score because to remove them could artificially inflate the proportion of good or excellent slices, which would be a misrepresentation of what was available with the investment of time to acquire all 16 scans. However, the statistical model used the exact number of scans used to estimate the relationship between HIA clarity and number of scans averaged. The presence of a shortened dataset due to movement was included in our multivariate logistic regression model and did not significantly affect the results when correcting for the number of scans used.

### Image Scoring

**FIGURE 2:**
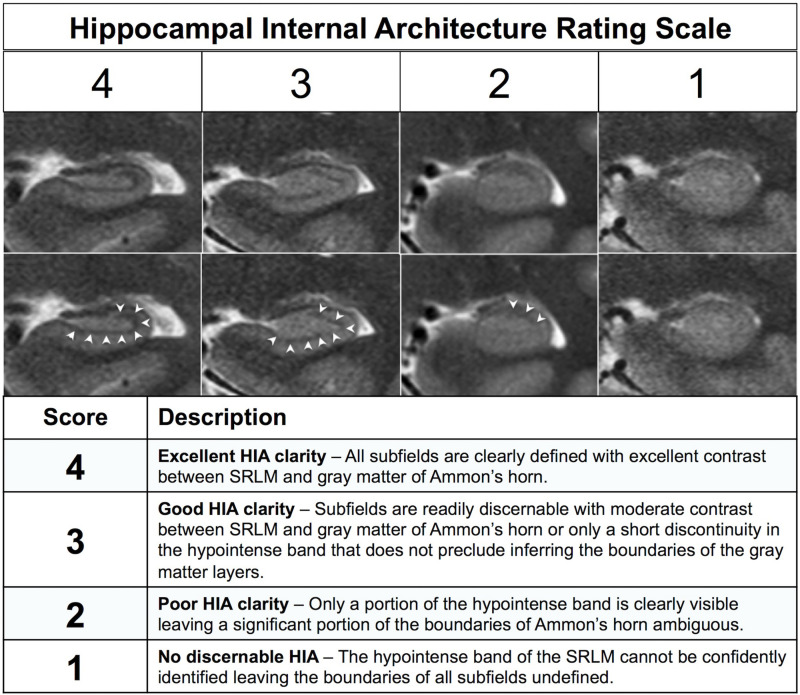
Description of the previously published HIA rating system. The images show examples of each of the four scoring levels. The both rows of images are identical except for the addition of arrowheads in the bottom row to indicate the dark band of the SRLM.

After generating separate mean images (HR-MICRA 4x, HR-MICRA 9x, and HR-MICRA 16x), each slice through the hippocampi was scored on each side for HIA clarity according to a previously published and validated rating scale (Ver Hoef et al., 2013a). This rating scale is based on clarity of visualization of the hypointense band of the SRLM allowing delineation of hippocampal subfields and is described in brief in Figure 2 (Ver Hoef et al., 2013a). Scoring was performed by one of two experienced raters (LWV, JC), both of whom were involved in a previous study describing the rating system and reporting its inter-rater reliability. Because inter-rater reliability was already established and published including these two raters (Ver Hoef et al., 2013a), it was not deemed necessary to repeat it within this dataset.

The anatomic differences of the head, body, and tail make imaging HIA more or less challenging in different segments, particularly the digitations of the head. Therefore each segment (head, body, and tail) was scored separately. Similar to other published hippocampal segmentation schemes (Boccardi et al., 2015), the landmark for the boundary between head and body was the first slice in which the hippocampus shows the characteristic c-shaped appearance and is not a double-layered structure, and boundary between body and tail was the first coronal slice showing in which the quadrigeminal cistern can be seen in the midline (i.e., the plane of the superior and inferior colliculi). In the most anterior slices through the head, the SRLM appears as a single horizontal line without distinct subfields, so scoring in the head started six slices posterior to the first slice in which the SRLM is visible. In the tail, scoring continued until the hippocampal gray matter abuts the splenium of the corpus callosum. Scores in the head, body, and tail are reported separately and together.

### Statistical Analysis

We used generalized estimating equations (GEE) for ordinal data (Heagerty and Zeger, 1996) to examine the association between HIA ratings of hippocampal architecture clarity and image type (4x HR-MICRA, 9x HR-MICRA, and 16x HR-MICRA) and side (left, right) for each slice. GEE was employed as it utilizes data from all participants and provides more robust estimates in the presence of missing data (Schafer and Graham, 2002). All analyses modeled the odds of higher ratings (better clarity) with the 4x HR-MICRA images used as the reference category. Since SNR increases with the square root of the number of averages, we modeled the relationship between number of averages and improvement in HIA clarity using the *square root* of the number of averages instead of as a direct linear relationship between the raw number of averages. An exchangeable correlation structure was used to model the correlation of scores among images obtained from the same participant. Analyses were performed using the *geepack* package (Halekoh et al., 2006), version 1.2-1, in the R statistical environment, version 3.5.0 (R Core Team, 2017).

## Results

**FIGURE 3:**
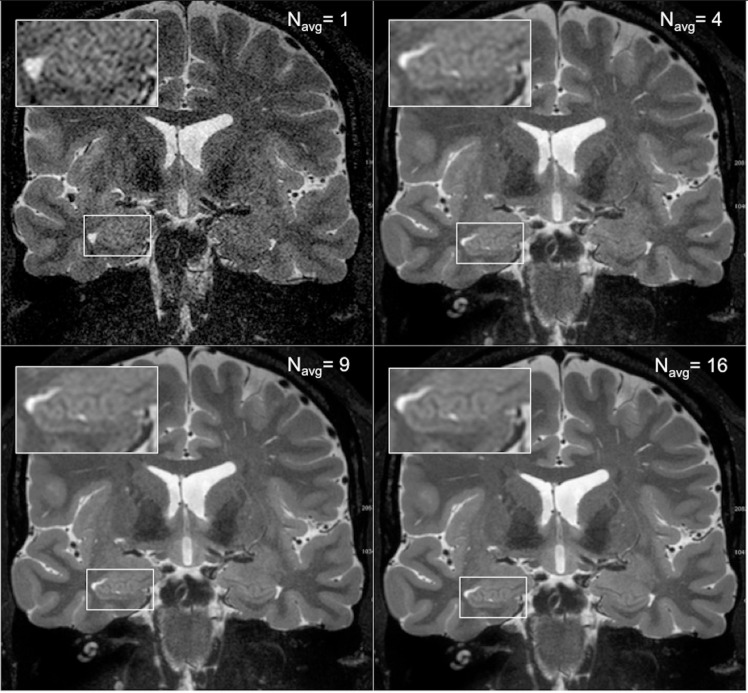
The four panels show how image quality improves with increasing the number of scans averaged. The **upper right panel** shows a single scan and the inset box shows that essentially no internal structure can be distinguished from background noise. The subsequent panels show the result of averages of 4, 9, and 16 samples, which corresponds to a boost of SNR by a factor of 2, 3, and 4, respectively, and increased clarity of the digitations of the hippocampal head.

**FIGURE 4:**
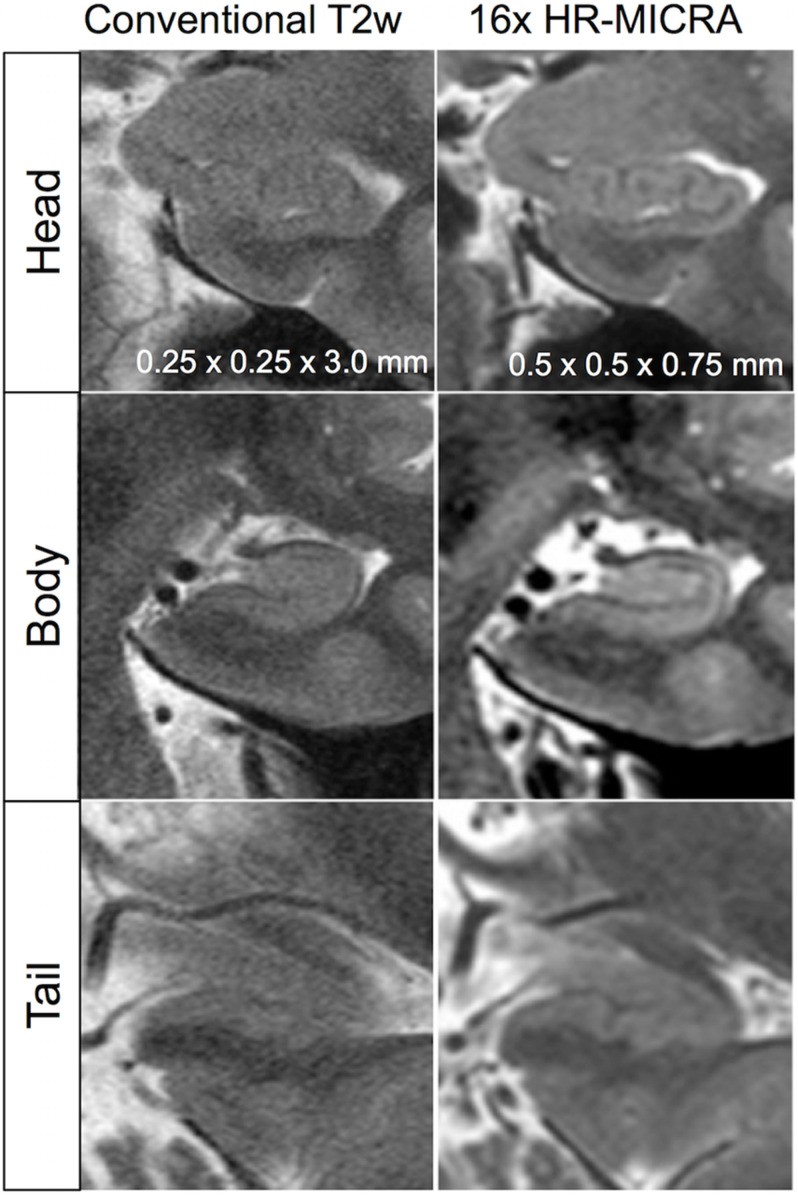
Illustration of differences in internal architecture clarity between standard T2w images and 16x HR-MICRA images across hippocampal segments in a single subject.

**FIGURE 5:**
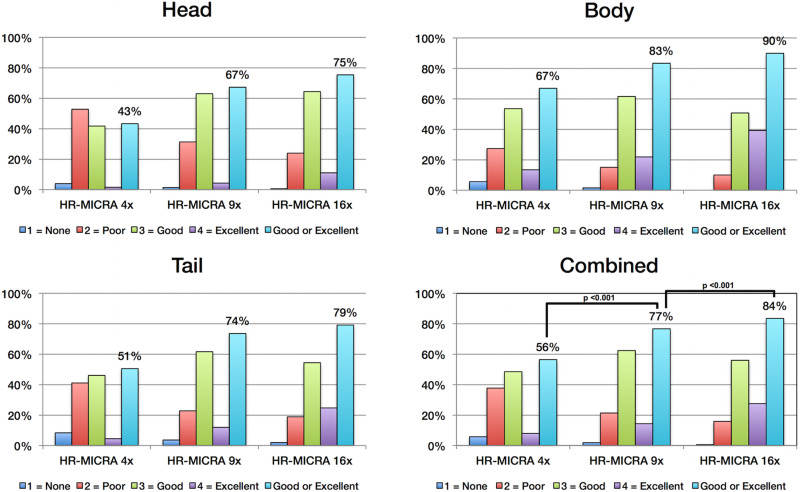
Charts show the distribution of HIA scores for each hippocampal segment and for all segments combined. Each bar represents the percent of slices across all subjects receiving the designated HIA score. The light blue colored bar indicates the percentage of slices scored as a 3 or 4, which is sufficient for direct visualization of all subfields. Statistically significant comparisons are marked with bars between pairs.

As expected, an obvious reduction in noise is visible with the increased number of samples averaged using the HR-MICRA approach. Figure 3 illustrates how image quality and HIA clarity improves from a single scan to HR-MICRA 4x, HR-MICRA 9x, and HR-MICRA 16x. Examples of HIA clarity in representative slices of each hippocampal segment are shown in Figure 4 depicting their markedly different cross sectional appearances. The distribution of HIA scores for the head, body, tail, and total hippocampus are shown in Figure 5. An HIA score of 3 or 4 indicates that all subfields can be directly visualized; therefore for purposes of doing accurate subfield segmentation, the proportion of slices rated as a 3 or greater is the best metric of performance. In the body of the hippocampus, the proportion of slices scoring 3 or greater is 90%, 83%, and 67% for the 16x, 9x, and 4x HR-MICRA images, respectively. Using the 4x HR-MICRA images as a baseline, the 9x HR-MICRA images were 2.6 times, and 16x HR-MICRA images were 3.2 times more likely to have high HIA ratings (*p* < 0.001). As expected, due to differences in the complexity of anatomy across the three segments of the hippocampus the data shows the head has 58% more low ratings (2 or less) than the body (*p* < 0.001) across all HR-MICRA images. The ratings for the tail segment were also lower, but this was not statistically significant (*p* = 0.06). No significant differences in HIA scores were seen between left and right sides (*p* = 0.627) across all image types.

**FIGURE 6:**
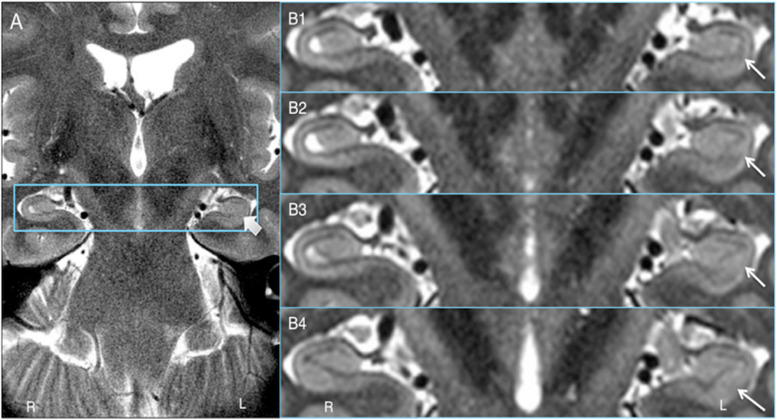
Volume averaging effects. **(A)** shows a 3 mm thick standard T2 slice and the thick arrow showing blurring of the SRLM. **(B1–4)** shows four contiguous 0.75 mm thick HR-MICRA slices that together cover the same 3.0 mm thick coronal slab as **(A)**. The thin arrows show that the contour of the SRLM on the left varies significantly from slice to slice in the inferolateral aspect of the hippocampus, which results in blurring in that portion of the SRLM in **(A)** due to volume averaging. The SRLM of the right hippocampus is generally consistent across slices in **(B1–4)** and is not blurred in **(A)**.

Key to demonstrating HIA clearly is obtaining slices that are thin enough to minimize volume averaging effect. A common 2D coronal T2w sequence used at our institution has an in-plane resolution of ∼0.25 mm and slices that are 3 mm thick versus the HR-MICRA images that are 0.75 mm thick, thus four HR-MICRA slices cover the same 3 mm thick slab of tissue represented by a single conventional T2 slice. As such, any feature of the image that is consistent across all four HR-MICRA slices will be represented well in a single conventional T2w slice, but any features that vary across those four slices will be blurred in the conventional T2w slice due to volume averaging. This is illustrated in Figure 6. Specifically, note that the lateral inferior portion of the SRLM in the conventional T2 slice is blurred, but the four HR-MICRA images that correspond to the same slab show that the contour of the SRLM is changing markedly in this area from slice to slice, even with a slice thickness of only 0.75 mm. By contrast, on the right side the SRLM has a more consistent appearance across three of the four HR-MICRA slices in the CA1 region, which translates to good HIA clarity in the corresponding conventional T2w image. It is also important to note that the HR-MICRA images in Figure 6 show the SRLM more clearly despite the fact that the *in-plane* resolution of the HR-MICRA images (0.5 mm) is significantly less than that of the conventional T2w images (0.25 mm).

**FIGURE 7:**
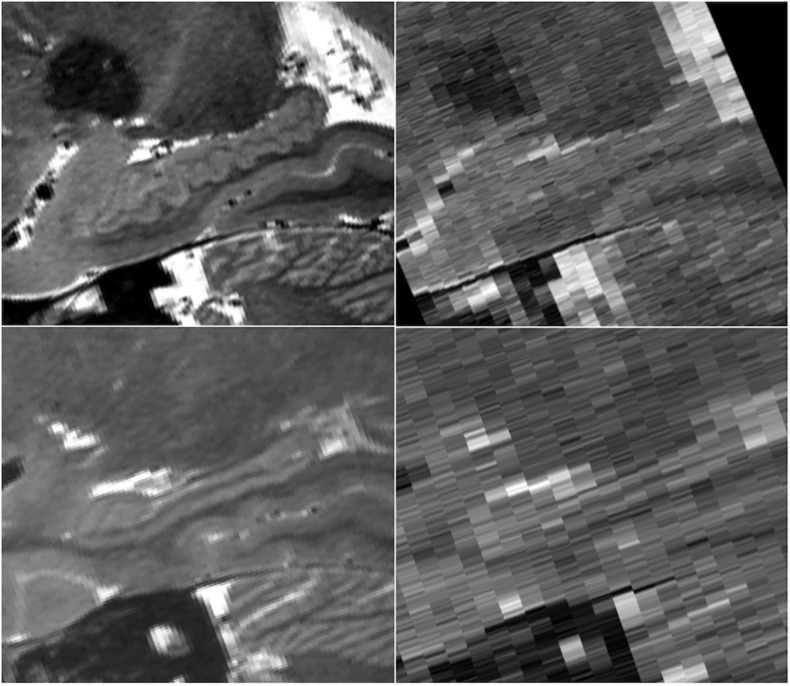
Sagittal views of a prominently dentated hippocampus **(top)** and minimally dentation hippocampus **(bottom)** using HR-MICRA 16x **(left)** and conventional T2 **(right)**. The difference in dentation between the upper and lower images is obvious in the HR-MICRA images, but the thick slices of the conventional T2 image do not allow differentiation between the two. Images are not interpolated.

Because the HR-MICRA approach is a 3D acquisition with a sub-millimeter slice thickness, the 3D volumes can be reformatted in any plane with good image clarity. This allows for clear visualization of hippocampal dentation, a morphologic feature of the human hippocampus that varies dramatically across healthy individuals and correlates with measures of memory (Beattie et al., 2017). While some individuals have rather “smooth” contours on the inferior aspect of the hippocampus, other individuals show a prominently dentated (tooth-like) appearance of the inferior aspect of the hippocampus as shown in Figure 7. Note that both of the individuals in this figure are healthy controls, so the variation in the degree of complexity of the hippocampal contours is not due to pathology. In contrast, all information about dentation is lost when conventional 2D approaches with thicker slices are used as shown in Figure 7.

**FIGURE 8:**
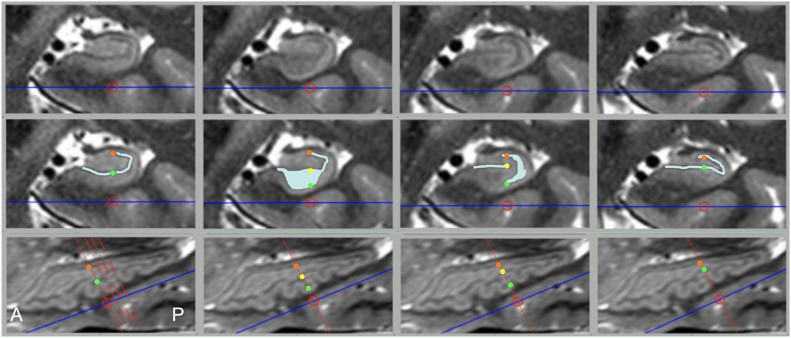
Dramatic changes in SRLM shape can be seen in consecutive slices in prominently dentated hippocampi. The **top row** shows coronal slices through a left hippocampus and **middle row** shows identical slices with the SRLM highlighted in light blue and index points marked with colored dots. The **bottom row** shows a sagittal slice through the same hippocampus. Solid red lines indicate the slice location of the images above, the dashed red lines in the bottom left image indicate the relative location of the other three slices, and the colored dots indicate the location of the corresponding dots in the **middle row**. In the first and last column, the SRLM has a simple curvilinear shape, but in the second column the SRLM is cut *en face* as it dips down into a dente (fold in CA1) and widens in appearance. In the third column, the SRLM is split into two parts as it weaves in and out of the plane of section.

While diagrams of the hippocampus and exemplary MRI images almost universally show SRLM to have a perfect C-shape (Figure 1), thin-sliced images can show a great deal of variation in the appearance of the SRLM, particularly in subjects with prominently dentated hippocampi. Figure 8 demonstrates how the SRLM can vary from being a thin band to a very thick band or even thin discontinuous segments in adjacent slices depending how it cuts through the folds in CA1 that create hippocampal dentation.

## Discussion

Many reports have been written about subfield segmentation but rarely is it stated how clearly the imaging sequence used showed HIA, or if it is mentioned, the ability of a particular sequence to show HIA is not examined rigorously. Instead it is generally merely presumed that HIA is shown well enough to outline subfields or at least to infer their location from surrounding landmarks. Similarly, automated subfield segmentation algorithms tend to rely heavily on atlas templates to apply probabilistic estimates of subfield boundaries to fill in gaps when the source image is not clear and may even segment a subfield that is not visible in the source image. In this work we evaluate HR-MICRA, an approach that allows direct visualization of the hippocampal internal architecture and hippocampal subfields and we rigorously examine its performance. We show HR-MICRA’s ability to delineate all subfields clearly in the hippocampal body in up to 90% of individual slices. Furthermore, the location of subfields in most of the other suboptimal slices may be reasonably estimated from adjacent slices because they are likely to have good clarity and with minimal interpolation error due to the small slice thickness.

Not only does this approach provide greater clarity of each slice, it provides many more slices and correspondingly much more data. Because the coronal slice thickness of HR-MICRA images is less than 1 mm, the image volumes are amenable to reformatting in any plane. This is best illustrated in visualizing the complex contours of hippocampal dentation in the sagittal plane, which would be impossible to visualize with common 2D approaches (Figure 7). Angulation of the plane of section such that it is orthogonal to the long axis of the hippocampus is important to optimize HIA clarity when slices are relatively thick, but for many subjects, particularly those with prominent dentation, slice thicknesses in the range of 2–4 mm are insufficient to avoid volume averaging effects, as seen in Figure 6. Ideally, the slice thickness should be relatively small compared to the contour of the structure to be visualized for volume averaging effects to be ameliorated, and contour information of features that vary significantly across a thick slice cannot be seen even with many averages. Dentation can be visualized to a limited degree in other 3D volumetric sequences, such as a T1w MPRAGE, but only when HIA is seen with sufficient contrast in the coronal plane can distinct gray and white matter layers be visualized in the sagittal plane. So, while dentation may be appreciated as bumps on the inferior surface in common T1w images, HR-MICRA images allow visualization of all the layers and the full extent of in-folding of CA1.

Thinner slices will also allow for more precise measurements of subfield volumes and surface contours, particularly in cases with prominent dentation where the subfield pattern varies significantly from slice to slice. Consistent and clear visualization of HIA should also allow automated or semi-automated subfield segmentation algorithms to more accurately define these small, but distinct, and important regions of interest by relying more on the features of the image and less on an atlas template. In this study, averaged images were created in the same resolution as the source images using linear registration methods for simplicity sake. However, image quality potentially could be further improved by upsampling images to an even higher resolution and/or employing non-linear registration techniques as has been demonstrated elsewhere (Shaw et al., 2019). While the ability to show HIA clearly with this approach is very good, it is not perfect. In fact, one in 10 of slices through the body show suboptimal HIA clarity, as well as one in four in the head and approximately one in five in the tail. However, these limitations should be considered in context. First, even with relatively thin slices of 0.75 mm, volume averaging effects are still at play for layers that dramatically undulate through the plane of section as seen in the second column of Figure 8. This phenomenon, particularly frequent in subjects with prominent dentation, is a common cause of blurring of HIA in the body and tail. This will always be a factor for visualizing HIA until slices become extremely thin (e.g., much less than 0.5 mm), which will be very challenging at 3T. Second, using a sub-millimeter slice thickness provides many more slices, which minimizes error that comes from inferring the location of an ambiguous subfield boundary. For example, if only one in ten or one in five slices shows suboptimal HIA out of 60 slices through a typical 4.5 cm long hippocampus, the contours of the subfield boundaries can be reasonably estimated from surround slices with minimal error. Furthermore, most of the suboptimal slices still show some degree of internal architecture [HIA rating of 2 (Figure 2)], and no slices in the body (for HR-MICRA 16x) and very small percent in the head and tail show no internal architecture at all. This means that using adjacent slices to infer the location of the SRLM, which defines HIA, is only necessary for *part* of the SRLM in almost all cases, which further minimizes error. With HR-MICRA, subfield definition in the body is excellent, which is the area focused on the most in the subfield segmentation literature. While HIA clarity is not as consistent in the head and tail as it is in the body, which is a nearly always the case in *in vivo* hippocampal imaging, our finding of good HIA clarity in 75%+ of slices in the head or tail is remarkable compared to standard T2 sequences with thick slices that virtually never show HIA clearly in the head and tail.

The obvious limitation of this approach is the amount of time it takes to acquire a full dataset. The 16x HR-MICRA scans take well over 1 h. However, several factors are important to consider. First, The approach is flexible and can be adapted to, however, much time is available. Even the 4x HR-MICRA images showed HIA significantly better than conventional T2 images. The repetition of the sequence up to 16 times here was not intended to be the most common implementation of this approach, but rather to demonstrate how good the performance can be if enough time is committed. The improvement in percentage of scans showing HIA clearly is roughly linear from 4 to 9 to 16 averages, which is consistent with the theoretical linear relationship between SNR and the square of the number of images averaged. As such, if the scan time did not allow acquiring four scans, one could extrapolate the SNR of averaging less scans, which would be proportional to the ratio of the square root of the number of scans averaged [e.g., SNR_3_/SNR_4_ = sqrt(3)/sqrt(4) = 0.87]. This yields an estimated 13% decrement in clarity using a three-scan average and 29% decrement using a two-scan average compared to a four-scan average. Likewise, the clarity for any higher number of scans could be estimated in a similar fashion. Second, the current experiments were performed on a first-generation clinical 3T scanner (installed 2004) with only an 8-channel parallel coil to demonstrate that high-resolution 3D data sets can be generated without the necessity of ultra high field scanners (7T+) or state-of-the-art high performance 3T scanners, which makes access to such high quality images far more widespread. Given enough time and repetitions, virtually any scanner worldwide could generate very high quality images with this approach, even with field strengths lower than 3T. We have, however, implemented a similar sequence on a Siemens Prisma platform (Siemens Healthcare, Erlangen, Germany) with a high performance gradient set and 64-channel head coil and are able to generate a 6x average image that is similar to the 16x HR-MICRA images in this study, for a time savings of almost two-thirds (data not shown). Higher channel-count coils (e.g., 32, 48, and 64) that are commonly available now allow both higher SNR and better parallel acceleration. Third, at this point this approach is not intended to be used as part of a routine clinical study, but rather as a research tool for investigating the hippocampus or other brain structures requiring high resolution in all three dimensions. Though, combining this approach with other highly accelerated MRI acquisition techniques, such as CAIPI imaging (Breuer et al., 2006; Bilgic et al., 2015) (Controlled Aliasing in Parallel Imaging Results in Higher Acceleration), other forms of simultaneous multi-slice imaging (Barth et al., 2016), or compressed sensing (Lustig et al., 2007; Toledano-Massiah et al., 2018) for example, may further compress the total study time to something reasonable for a research protocol. In any case, we want to be very clear that this approach is best suited for applications that place a premium on hippocampal internal architecture clarity and can justify investing significant scanner time to obtain high resolution hippocampal images and is not intended to be used in routine clinical scans.

Numerous factors affect SNR including TE, TR, acceleration factor, acquisition scheme (2D vs. 3D, constant refocusing flip angle vs. variable flip angle), and many others. And as described in the “Materials and Methods” section, we deliberately chose a short TR in order to get more TRs in limited time to scan the whole volume, but the short TR limits the relaxation between excitations and consequently decreases the SNR because there is less longitudinal magnetization to be excited at the beginning of the next TR. However, this short TR allows scanning of a large, high-resolution volume in a reasonable period of time (which minimizes movement) with good gray-white contrast, at the expense of SNR. SNR can be compensated for with co-registration and averaging of additional acquisitions, but intra-scan movement and poor gray-white contrast that come with longer sequences with better SNR cannot.

There is a slight smoothing effect that comes from interpolation in resampling a co-registered image volume. This effect is more prominent when the source image and target image have different resolutions. Since our images were all of identical resolution, we did not consider this to be a significant factor and any smoothing that may be present was not apparent in visual comparison of the original reference image to the co-registered images. A nearest neighbor interpolation scheme would avoid any smoothing, but is not commonly used. Moreover, since the approach relies heavily on averaging anyway the effect of smoothing from interpolation is expected to be comparatively small.

## Conclusion

High Resolution Multiple Image Co-registration and Averaging is a flexible approach that provides consistently clear visualization of HIA that is notably inconsistent in common 2D TSE scans. Using thin coronal slices not only minimizes volume averaging effects but also allows reformatting in any plane of section, which allows for study of hippocampal morphologic features such as dentation. The improved clarity of HIA visualization allows more precise and accurate segmentation of hippocampal subfields as well as more sensitive detection of pathologic alterations of HIA. This approach was demonstrated using a basic 3T imaging platform without advanced head coils, advanced pulse sequences, or proprietary processing software, making it widely accessible to any imaging laboratory. Furthermore, the approach is generally applicable to and will further benefit from advanced imaging techniques like ultra high field imaging, high channel head coils, and accelerated pulse sequences and reconstruction schemes.

## Data Availability Statement

Data used in the study will be made available to investigators upon request after receipt of an IRB approved protocol and a signed data use agreement from the receiving institution.

## Ethics Statement

The studies involving human participants were reviewed and approved by the Institutional Review Board, The University of Alabama at Birmingham, Birmingham, AL, United States. The patients/participants provided their written informed consent to participate in this study.

## Author Contributions

LV: conceptualization, formal analysis, funding acquisition, investigation, methodology, project administration, supervision, roles/writing – original draft, and writing – review and editing. HD: data curation and investigation. JC: formal analysis. GS: data curation and writing – review and editing. JB: data curation, investigation, and writing – review and editing. REK: formal analysis, methodology, software, and writing – review and editing. RCK: conceptualization, methodology, and writing – review and editing. JS: conceptualization and writing – review and editing. All authors contributed to the article and approved the submitted version.

## Conflict of Interest

The authors declare that the research was conducted in the absence of any commercial or financial relationships that could be construed as a potential conflict of interest.
