# Supplementary material for: Clear and Consistent Imaging of Hippocampal Internal Architecture With High Resolution Multiple Image Co-registration and Averaging (HR-MICRA)
Source: Front Neurosci. 2021 Feb 11;15:546312. doi: 10.3389/fnins.2021.546312 (PMC7905096; doi:10.3389/fnins.2021.546312)
Supplement: Supplementary file 1 [file Data_Sheet_1.PDF]

Supplemental information

Coregistration and averaging in this work used FSL, which can be found at https://fsl.fmrib.ox.ac.uk/fsl/fslwiki/FslInstallation

Three commands in the FSL library are used:

1. *flirt* is used to coregister each of the image volumes for a given subject to the first image volume for that subject.
2. *fslmerge* is used to combine all of the coregistered image volumes for a given subject into a single 4D file.
3. *fslmaths* is used to create a single mean image volume from the coregistered image volumes in the merged file for each subject.

The bash shell script below can be used to generate the coregistered images and the mean image. It assumes that the image volumes are organized into folders named “subjXX” (e.g. subj01, subj02, … ; subj_001, subj_002, … ; or subj_A, subj_B, … etc) for each subject, with individual image volumes in those folders named “hrmicra_run01.nii.gz”, “hrmicra_run02.nii.gz”, etc. This also assumes that all the subject folders are in a folder named “HRMICRA” in the path ‘/Users/*username*/Documents/’. This script will process all subjects in the HRMICRA folder in a single execution and produce a coregistered and averaged image named “hrmicra_mean.nii.gz” in each subject folder. Image volumes are assumed to be in compressed NIfTI format (nii.gz). A tool to convert DICOM images to NIfTI format can be found at https://people.cas.sc.edu/rorden/mricron/dcm2nii.html

```
#!/bin/bash
homedir='/Users/username/Documents/HRMICRA'
cd $homedir;
export FSLOUTPUTTYPE=NIFTI_GZ;
        for subs in `ls -d subj*`; do                    #Loop across all the participants

                for files in `ls hrmicra_run*.nii.gz | grep -v 01`; do        #Loop through all files except the first one

                        export targ=`hrmicra_run01.nii.gz        #First run is the template/target
                        export coarse=60;
                        export cost=mutualinfo;
                        export dof=12;
                        clear;

                        mov=$files;
                        out=`echo $mov | cut -d '.' -f1`;
                        out=`printf "%s_coreg" $out`;            #Name the output files _coreg
                        omat=transform.mat;
                        cmd=`echo -e "flirt -in $mov -ref $targ -out $out -omat $omat -dof $dof -cost $cost -coarsesearch $coarse"`;
                        echo $cmd
                        eval $cmd;
                done

        # Combine all the registered images and template image in a single 4D file
        fslmerge -t hrmicra_all `ls *_coreg.nii.gz` hrmicra_run01.nii.gz    # Combined file is named hrmicra_all.nii.gz

        fslmaths hrmicra_all.nii.gz -Tmean hrmicra_mean                  # Average the 4D files across "time" to get a mean image hrmicra_mean

        done
```

Notes:

- If the user prefers to do coregistration and averaging without the bash script, the 2nd through nth images can be coregistered to the 1st one by one using the following command repeatedly for each image *hrmicra_runXX*:
  flirt -in *hrmicra_runXX* -ref hrmicra_run01 -out *hrmicra_runXX*_coreg -dof 12 -cost mutualinfo -coarsesearch 60
- If the coregistration step fails to accurately coregister the images, try using a different cost function (i.e -cost leastsq or -cost corratio instead of -cost mutualinfo)
- The set of coregistered images can be merged into a single 4D file called hrmicra_all using the following command:

fslmerge –t hrmicra_all hrmicra_run_01.nii.gz hrmicra_run02_coreg.nii.gz hrmicra_run03_coreg.nii.gz … hrmicra_run16_coreg.nii.gz

- The merged images can be averaged into a mean image using the following command:

  fslmaths hrmicra_all.nii.gz -Tmean hrmicra_mean
